# Granulomatous Slack Skin: A Case Report

**DOI:** 10.5826/dpc.1002a44

**Published:** 2020-04-03

**Authors:** George Balais, Aimilios Lallas, Elizabeth Lazaridou, Linda Kanatli, Zoe Apalla

**Affiliations:** 1 First Dermatology Department, Aristotle University of Thessaloniki, Greece; 2 Second Dermatology Department, Aristotle University of Thessaloniki, Greece; 3 Dermatology Department, Bozova National Hospital, Bozova Sanliurfa, Turkey; 4 State Dermatology Department, Hippokratio General Hospital, Thessaloniki, Greece

**Keywords:** granulomatous slack skin, mycosis fungoides, T cell, lymphoma, lymphocytes

## Introduction

Granulomatous slack skin (GSS) is a very rare subtype of mycosis fungoides (MF), with fewer than 50 cases already reported. It is 1 of the 3 variants of MF, along with folliculotropic MF and pagetoid reticulosis (World Health Organization-European Organization for Research and Treatment of Cancer, 2018). There is a male predominance and an onset usually during adulthood.

## Case Presentation

**Figure 1:**
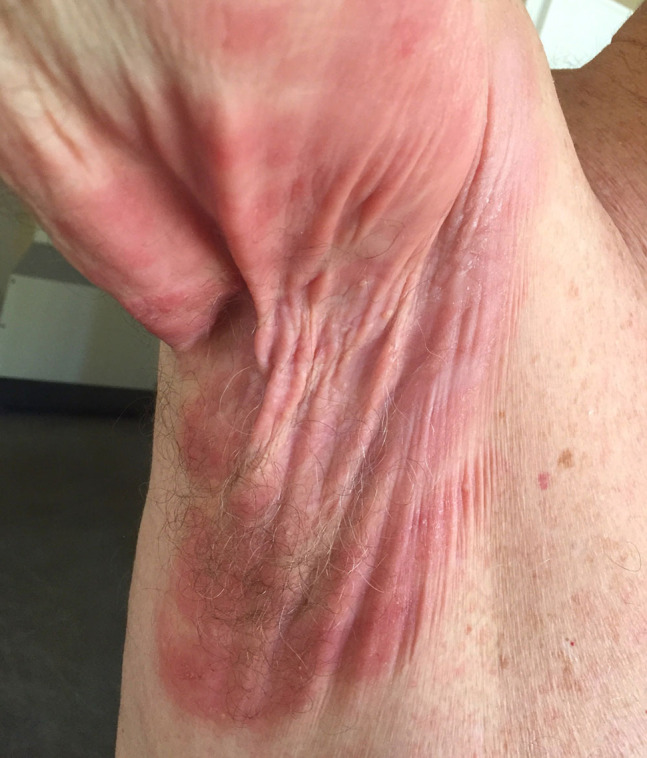
A well-circumscribed erythematous plaque, consisting of pendulous, wrinkled, lax skin, occupying the axilla is typical of granulomatous slack skin.

**Figure 2:**
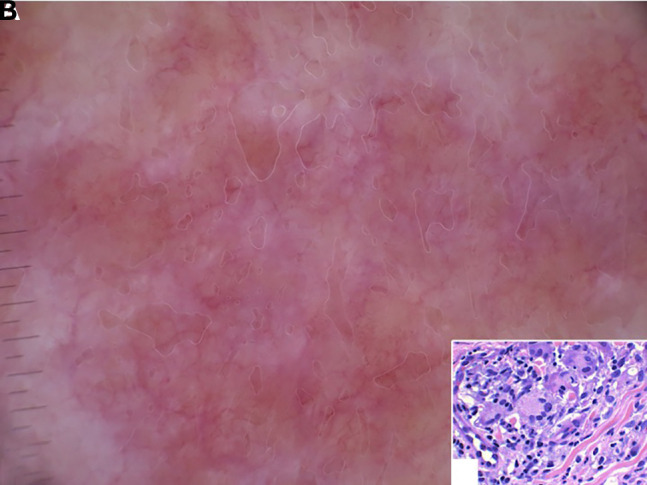
(A) The dermoscopic pattern consisting of pale orange areas on an erythematous background together with fine linear vessels is indicative of a granulomatous skin disease. (B) Dermal infiltrate consisting of atypical, irregular, convoluted lymphocytes, together with multinucleated giant cells (H&E, ×40).

A 74-year-old man presented with a 4-year history of a lesion on his right axilla. During these years the patient had received various diagnoses, including intertrigo, dermatitis, and inverse psoriasis. Based on his experience, minimal remission was observed only with the use of a medium-potency topical steroid, but the lesion relapsed after treatment discontinuation. Clinical examination revealed a well-circumscribed erythematous plaque, consisting of pendulous, wrinkled, lax skin, unilaterally affecting the axillary fold (Figure 1). Dermoscopy showed pale orange areas on an erythematous background and fine linear vessels (Figure 2A), which were suggestive of a granulomatous dermatosis. GSS was our preferred diagnosis, owing to the striking clinical picture and the dermoscopic findings. Histological examination following a biopsy revealed a dense granulomatous infiltrate in the dermis, composed of atypical lymphocytes, macrophages, and giant cells (Figure 2B), along with a loss of elastic tissue. The epidermis was infiltrated by small, atypical lymphocytes with cerebriform nuclei. The atypical T cells had a CD3+, CD4+, CD7−, CD8− phenotype. The latter findings, as well as the monoclonal rearrangement of the T cell receptor genes, led to the diagnosis of GSS. Atypical lymphocytes were not present in the peripheral blood smear. Full-body CT scan and the rest of the laboratory investigation results were unremarkable. The patient partially responded to re-PUVA treatment and then he was referred for radiotherapy.

## Conclusions

GSS is clinically characterized by slow, progressive development of large areas of asymptomatic, pendulous, lax skin in the major skin folds, with a predilection for axillae and groins. Clinically, differential diagnosis includes acquired cutis laxa, anetoderma, as well as other dermatoses involving the skin folds [1]. To our knowledge, dermoscopic features of GSS have never been described in the literature. As expected for a granulomatous process, the dermoscopic clues included orange areas and fine linear vessels. The latter pattern is not diagnostic, but it is suggestive of histological presence of granulomas in the dermis.

The histological analysis reveals a dense, diffuse dermal infiltrate of atypical, irregular, convoluted lymphocytes (with a CD3+, CD4+, and CD8− phenotype) that may extend to the subcutaneous tissue, together with diffuse, multinucleated giant cells and histiocytes that display prominent elastophagocytosis and lymphophagocytosis. The granulomatous infiltrate is correlated with simultaneous loss of elastic fibers. Molecular analysis reveals a monoclonal rearrangement of the T cell receptor genes. The diagnosis of GSS, as in our case, is based on the typical clinical and histological features [2].

The disease follows an indolent course, with an excellent prognosis. The reported 5-year survival rate is almost 100%. However, since there is an association with other lymphoid neoplasias (mainly with Hodgkin lymphoma), lifelong monitoring is recommended [2].

There is no standard of care for GSS. Common therapeutic strategies are PUVA, local or total skin electron beam radiotherapy, nitrogen mustard, interferons, corticosteroids, retinoids, azathioprine, methotrexate, or chemoimmunotherapy [2]. Surgical excision can be considered, but it is only palliative, and recurrences usually occur [1].
